# Effect of lymph node resection on prognosis of resectable intrahepatic cholangiocarcinoma: A systematic review and meta-analysis

**DOI:** 10.3389/fonc.2022.957792

**Published:** 2022-09-27

**Authors:** Feiyu Li, Yong Jiang, Liyong Jiang, Qingbin Li, Xiangyu Yan, Songhan Huang, Ji Chen, Shuai Yuan, Yingda Fu, Jun Liu

**Affiliations:** ^1^ Shandong Provincial Hospital, Shandong University, Jinan, China; ^2^ Shandong Provincial Hospital, Shandong First Medical University, Jinan, China

**Keywords:** intrahepatic cholangiocarcinoma, lymph node, prognosis, overall survival, disease-free survival

## Abstract

**Background:**

The purpose of this meta-analysis was to evaluate the efficacy of lymph node dissection in patients with intrahepatic cholangiocarcinoma (ICC).

**Methods:**

The literature from January 2009 to December 2021 was searched to determine the comparative study of lymph node dissection and non-lymph node dissection in patients with ICC.

**Results:**

Seventeen studies were included in the analysis. There were no significant differences in 1-, 3-, and 5-year overall survival (OR = 0.80, p = 0.10; OR = 0.93, p = 0.71; OR = 0.80, p = 0.21) and 1-, 3-, and 5-year disease-free survival (OR = 0.89, p = 0.73; OR = 0.92, p = 0.81; OR = 0.85, p = 0.62).

**Conclusions:**

Lymph node dissection does not seem to have a positive effect on the overall survival and disease-free survival.

## Introduction

Intrahepatic cholangiocarcinoma (ICC) is a kind of primary liver cancer, and its incidence is second only to hepatocellular carcinoma. In particular, ICC accounts for 8%–10% of biliary tract cancers and 10%–20% of all primary liver tumors (1). Worldwide, the overall incidence of intrahepatic cholangiocarcinoma is on the rise (2). According to this trend, people have a growing interest in the management of ICC.

The onset of cholangiocarcinoma is hidden, and most patients are in advanced stage when the disease is found. Most patients with ICC are not candidates for curative resection because of advanced cancer at the time of initial presentation or underlying comorbidities (3). In the last decade, notable efforts have been made by the medical community in an attempt to improve clinical outcomes of patients with unresectable ICC with the development of various treatment methods, including immunotherapy (4), targeted therapy (5), chemotherapy (6), transarterial radioembolization, hepatic artery infusion, transarterial chemoembolization, and radiofrequency ablation (7, 8).{Brandi, 2020 #4;Massa, 2020 #7;Rizzo, 2021 #5;Rizzo, 2021 #6;Sommer, 2016 #3}{Brandi, 2020 #4;Massa, 2020 #7;Rizzo, 2021 #5;Rizzo, 2021 #6;Sommer, 2016 #3}{Rizzo, 2021 #5}{Rizzo, 2021 #5}{Rizzo, 2021 #5}{Brandi, 2020 #4;Massa, 2020 #7;Rizzo, 2021 #5;Rizzo, 2021 #6;Sommer, 2016 #3}{Brandi, 2020 #4;Massa, 2020 #7;Rizzo, 2021 #5;Rizzo, 2021 #6;Sommer, 2016 #3} A careful evaluation of tumor burden appears as a crucial element in choosing the best therapeutic strategy in unresectable ICC. For those with resectable disease, surgical resection is the best choice (9). However, the prognosis of ICC is not ideal. There are several independent factors associated with the worse long-term survival rate, including the presence of vascular invasion, symptomatic disease, regional lymph node metastasis, and multiple tumors (10).

It is reported that LNM is one of the most prominent adverse prognostic factors in patients with ICC (11), but the role of lymph node dissection (LND) in resectable ICC is still controversial. A recent guideline recommended that regional lymphadenectomy should be performed in patients undergoing resection (12). However, evidence of the benefits from lymphadenectomy does not seem sufficient (13). Therefore, it is difficult to reach a consensus on whether LND should be performed routinely. In this study, therefore, a meta-analysis of the published literature was performed to assess the effect of LND on prognosis for patients with resectable ICC.

## Methods

The study protocol was published on PROSPERO, the international prospective register of systematic reviews (reference: CRD420223257). The search and analysis were performed according to the PRISMA (Preferred Reporting Items for Systematic Reviews and Meta-Analyses) Statement (14) and Cochrane Handbook for Systematic Review of Interventions (15).

### Literature search and selection criteria

The online databases of PubMed/Medline, Cochrane Library, EMBASE, and Web of Science were searched for all levels of evidence published in print or electronically from January 2009 to December 2021. Search terms contain “intrahepatic cholangiocarcinoma”, “cholangiocarcinoma”, “lymph node excision”, “lymph node dissection”, and “lymphadenectomy”. The included articles have no language restrictions.

Only articles that meet the following conditions will be included: (1) population: patients were pathologically diagnosed as cholangiocarcinoma and underwent surgery; (2) intervention: lymph node dissection (LND+); (3) comparison: no lymph node dissection (LND−); (4) outcome: 1-, 3-, and 5-year overall survival (OS) and 1-, 3-, and 5-year disease-free survival (DFS); and (5) design: comparative studies, including retrospective and prospective investigations.

Articles were excluded if patients were diagnosed pathologically as hepatocellular carcinoma, mixed type ICC, or cancer in other parts of the bile duct; the information provided in the article was non-comparable or insufficient for data extraction or quality assessment; and the articles were conference abstracts, letters to the journal editors, and review articles.

### Study selection

One investigator (Li F) performed the searching, inclusion, and exclusion of articles, which was subsequently double-checked by all other involved authors.

### Data extraction

Two independent researchers (Li F and Yan) reviewed the studies; disagreements in eligibility, data extraction, and quality assessment were resolved through discussion and consultation by all involved authors. Data extracted from each article included the first author, year of publication, study design, number of patients, matching criteria, and reasons for and area of LND. The primary outcome was 1-, 3-, and 5-year overall survival and 1-, 3-, and 5-year disease-free survival. Data after matched were extracted if patients were matched in studies.

### Quality assessment

The methodological quality of the observational studies was assessed using the Newcastle–Ottawa scale (NOS) (16). This assessment scale consists of three factors: patient selection, comparability of groups, and assessment of outcomes. A score of 0–9 was allocated to each study, which was estimated by two independent reviewers. Disagreements were resolved through discussion and consultation by all involved authors. Observational studies with a score of 6 or more were considered to be of high quality.

### Statistical analysis

Patients were divided into two groups according to whether lymph nodes were removed or not: non-lymph node dissection (LND−) group and lymph node dissection (LND+) group. Data analyses were performed using the Review Manager (RevMan, version 5.4.1) in accordance with the PRISMA guidelines. All variables were presented as dichotomous data to evaluate odds ratio (OR) and 95% confidence interval (CI). The OR of <1 favored the LND− group. P-value <0.05 was considered statistically significant. Statistical heterogeneity among studies was assessed using the χ² and I² statistics. A random-effects model was used if the heterogeneity among studies was considered present (P < 0.1 or I² > 50%). Otherwise, a fix-effects model was used.

## Results

### Study search results

**Figure 1:**
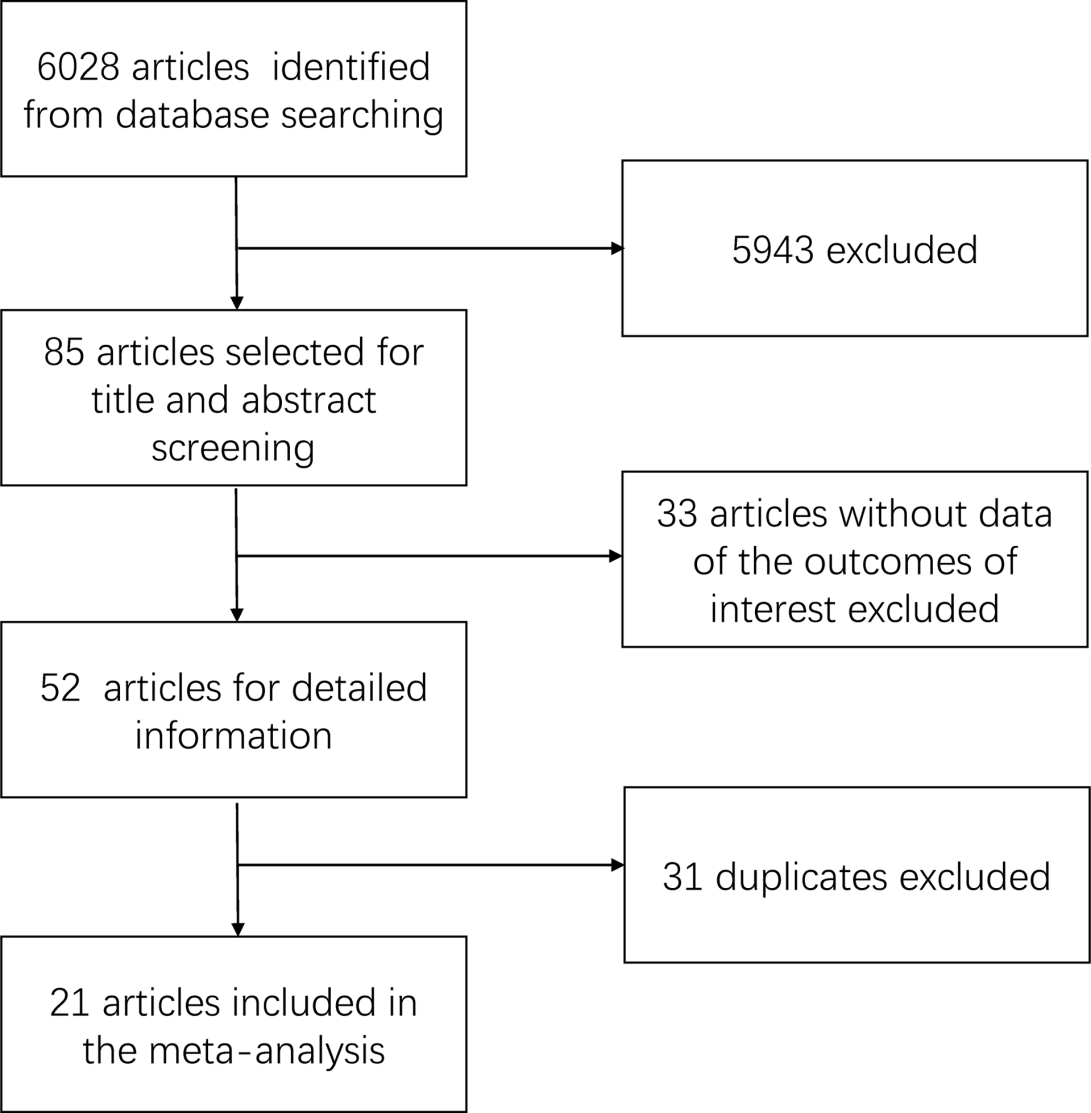
Diagram of literature search and study selection.

The summary of the search result is shown in **Figure 1**. We retrieved a total of 6,028 related articles. After preliminary screening, 85 articles met the inclusion criteria. We further excluded 33 articles because of the absent data in the outcome of interest. Finally, 31 duplicates were excluded. A total of 21 articles were included in this study, which included 3,796 patients (1,990 patients with LND+ and 1,806 patients with LND−).

### Characteristics of eligible studies

The included studies (17–37) were all retrospective cohort trials, and the publication dates ranged from 2009 to 2021. Eighteen studies (17–27, 30, 32–35, 37) were from East Asia, two studies (28, 29) were from Europe, one study (31) was from the US, and one study (36) was from Europe and East Asia. Seven multi-center studies (20, 23, 29–32, 36) were included. The largest multi-center study (31) involved 402 patients and used the Surveillance, Epidemiology, and End Results (SEER) database.

Three studies (22, 23, 32) had an overlapping population in the same center; therefore, only the study (32) with the largest time scope was included in the analysis. Two studies (19, 37) collected data in the same center, and there is an overlapping population in a certain part of the time. We only took the study (37) with a large number of patients into the analysis. Two other studies (26, 33) had the same condition, and only the study (26) with a relatively large number of patients was analyzed similarly.

### Survival outcomes

**Figure 2:**
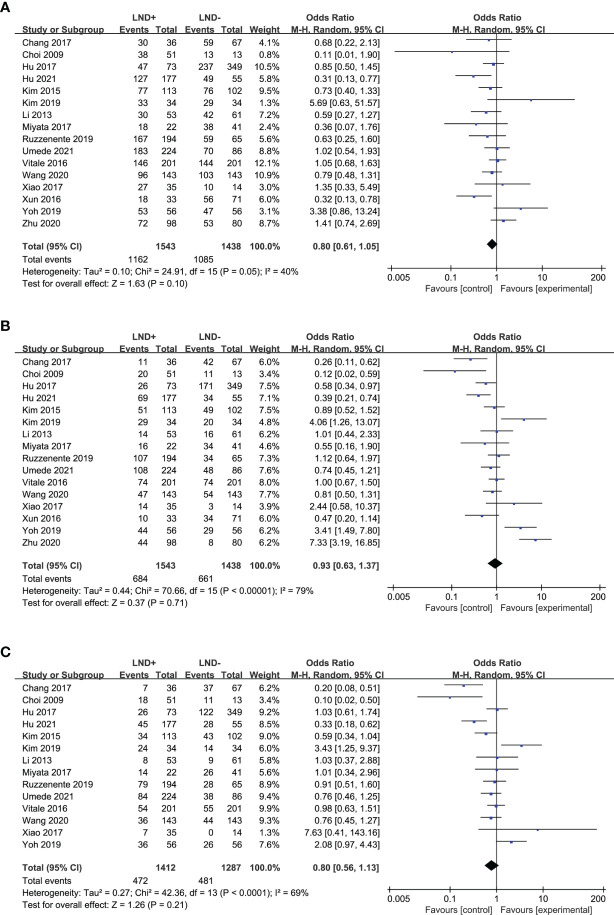
Forest plot comparing overall survival in LND+ and LND− groups. **(A)** 1-year OS; **(B)** 3-year OS; **(C)** 5-year OS.

Sixteen studies (17, 18, 20, 21, 24–27, 29–32, 34–37) were used to compare the 1- and 3-year OS of LND+ group and LND− group in the meta-analysis (**Figures 2A, B**). There was no significant difference between the two groups. Fourteen studies (17, 18, 20, 21, 24–27, 29–32, 34, 36) compared the 5-year OS of LND+ group and LND− group (**Figure 2C**). There was no significant difference between the two groups.

**Figure 3:**
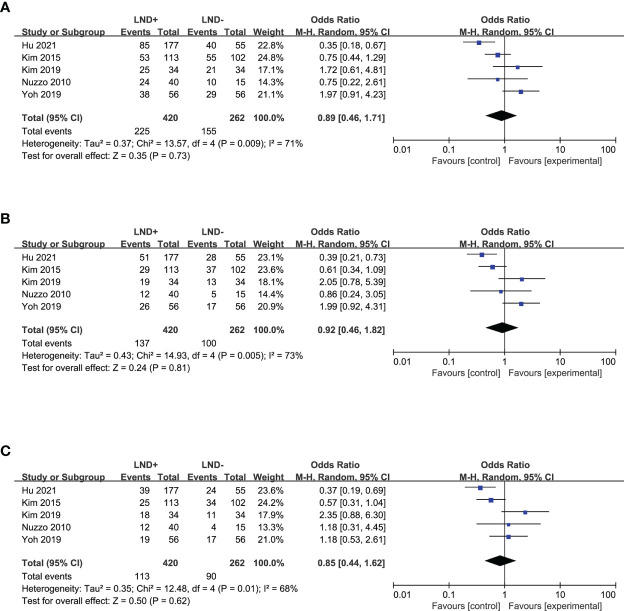
Forest plot comparing disease-free survival in LND+ and LND− groups. **(A)** 1-year DFS; **(B)** 3-year DFS; **(C)** 5-year DFS.

In a term of recurrence, six studies (20, 22, 24, 25, 28, 36) allowed for pooling of the data to obtain the 1- and 3-year DFS (**Figures 3A, B**), which showed no significant difference between the LND+ and LND− groups. Five studies (20, 24, 25, 28, 36) that compared 5-year DFS between the LND+ and LND− groups were suitable for data merging (**Figure 3C**). There was no significant difference between the two groups.

The sensitivity analysis result is shown in **Table 1**. Greater sample sizes (20, 21, 24, 29–32), multi-center (20, 29–32, 36), East Asia region (17, 18, 20, 21, 24–27, 30, 32, 34, 35, 37), high quality studies that score ≥6 on the NOS (21, 25, 26, 28, 31, 32, 36), and the publication date after 2018 (20, 25, 29, 30, 32, 36, 37) were assessed, respectively.

**Table 1:** Sensitivity analysis among groups.

	Studies	Patients(LND+/LND−)	Total	OR	P-value	Study heterogeneity	
						I²	p
Studies with sample size greater than 200							
1-year OS	7	1,125/1,001	2,126	0.81	0.05	9%	0.36
3-year OS	7	1,125/1,001	2,126	0.78	0.01	35%	0.16
5-year OS	7	1,125/1,001	2,126	0.76	0.007	43%	0.10
1-year DFS	2	290/157	447	0.52	0.01	69%	0.07
3-year DFS	2	290/157	447	0.50	0.001	3%	0.31
5-year DFS	2	290/157	447	0.47	0.0007	0%	0.32
Multi-center studies							
1-year OS	6	995/606	1601	0.85	0.45	51%	0.07
3-year OS	6	995/606	1601	0.94	0.76	73%	0.002
5-year OS	6	995/606	1601	0.82	0.33	66%	0.01
1-year DFS	2	233/111	344	0.82	0.81	91%	0.0008
3-year DFS	2	233/111	344	0.82	0.81	91%	0.0008
5-year DFS	2	233/111	344	0.64	0.45	80%	0.02
Studies from East Asia							
1-year OS	13	1,092/1,116	2208	0.74	0.04	36%	0.09
3-year OS	13	1,092/1,116	2208	0.82	0.40	79%	<0.00001
5-year OS	13	1,092/1,116	2208	0.69	0.01	70%	0.0002
1-year DFS	3	324/191	515	0.71	0.39	72%	0.03
3-year DFS	3	324/191	515	0.72	0.44	75%	0.02
5-year DFS	3	324/191	515	0.73	0.51	80%	0.008
High-quality studies (score ≥ 6)							
1-year OS	6	381/684	1065	0.87	0.38	44%	0.11
3-year OS	6	381/684	1065	1.20	0.48	74%	0.002
5-year OS	6	381/684	1065	1.19	0.37	50%	0.08
1-year DFS	3	130/105	235	1.56	0.11	0%	0.43
3-year DFS	3	130/105	235	1.72	0.05	0%	0.49
5-year DFS	3	130/105	235	1.47	0.17	0%	0.53
Studies published after 2018							
1-year OS	7	926/519	1445	0.97	0.91	58%	0.03
3-year OS	7	926/519	1445	1.49	0.26	87%	<0.00001
5-year OS	7	926/519	1445	0.96	0.88	77%	0.0005
1-year DFS	3	267/145	412	1.02	0.97	85%	0.001
3-year DFS	3	267/145	412	1.13	0.84	85%	0.001
5-year DFS	3	267/145	412	0.96	0.94	82%	0.004

OR, odds ratio; OS, overall survival; DFS, disease-free survival; LND, lymph node dissection.

## Discussion

This study quantitatively analyzed the effectiveness of LND in patients with resectable ICC. All of these patients were treated with radical surgery. Compared with patients who did not receive LND, patients who received LND had no survival benefit. Meanwhile, LND is not effective in reducing the probability of recurrence. In the sensitivity analysis to evaluate the number of patients included, a significant difference in support of LND− group was found. However, because of the significant differences between the two groups in baseline in these studies, these results should be carefully interpreted.

Comprehensive treatment based on surgery was recognized as the best mode to treat ICC, but the tumor recurred and metastasized early postoperatively, and the long-term survival rate is poor (38). The majority of studies considered that lymphatic metastasis was the most important prognostic determinant (13, 39, 40). The prevalence of LNM in ICC is as high as 17%–39.1% (41). Some surgeons point out that LND can improve ICC survival. However, some researchers have found that LND is only a staging operation, which has little effect on the prognosis (42, 43). The current findings suggest that LND may not prolong the survival of patients with ICC. The reasons may be as follows: (1) Patients mostly had intrahepatic recurrence after operation. If intrahepatic metastasis is not intervened at the same period, then it is difficult to effectively improve the prognosis of patients by LND alone (2). Once lymph node metastasis occurs, it indicates that the condition is late, and the lymph node metastasis may be beyond the scope of surgical dissection. Even if the scope of dissection is expanded, the effect is limited (44, 45).

Meanwhile, the extent of LND is still controversial. The 2021 NCCN Guidelines (12) and the consensus on American Association of Hepatobiliary and Pancreatic Surgery (9) recommend routine hilar lymph node dissection. In general, conventional or standard LND usually refers to the removal of lymph nodes along the hepatoduodenal ligament, and the area includes lymph node in lesser curvature or left gastric artery, when the tumors are located in the left lobe of the liver. The eighth edition of the AJCC cancer staging system (46) suggests routine LND and removal of at least six LNs. This system also clearly defines regional LNs. In addition to hilar nodes (common bile duct, hepatic artery, portal vein, and cystic duct nodes), regional LNs include the inferior phrenic and gastrohepatic lymph nodes in the left liver lobe. The right lobe covers the periduodenal and peripancreatic LN areas. However, the liver has multiple lymphatic drainage pathways, so further research is needed, possibly including the concept of sentinel lymph nodes.

As the results of surgical treatment of ICCs are generally poor, more and more attention has recently been paid to adjuvant therapy recently to further improve the surgical prognosis of ICC. While the clinical benefits of adjuvant therapy for ICC are still unclear, BILCAP randomized trial recently reported that capecitabine, an adjuvant, can improve the overall survival for biliary tract cancer (47). The potential survival benefits of adjuvant chemotherapy may be related to tumor subtypes, such as lymph node metastasis and the advanced tumor (48). In this perspective, LND is necessary to identify the node status.

The meta-analysis has several limitations. The included studies were almost retrospective, involving selection bias and data missing. The overall number of patients is low, which can easily lead to bias. The sample size of the study is not enough to offset the possible impact of individual differences on the results of the study, and there may be selective bias in the samples and treatment methods. Most of the included studies did not match the propensity score, and the baseline of patients was different. At the same time, some patients with ICC may have adopted other treatment methods such as radiotherapy, chemotherapy, or some molecular targeted drugs after surgery, and the influence of the treatment process on the research was not considered. All of these have led to the deviation and significant heterogeneity of the research results. The differences in patients’ physiological conditions, surgical methods and skills, indications and scope of LND, pathological characteristics of tumors, perioperative adjuvant therapy, and other factors may be the reasons for high heterogeneity.

In conclusion, the current evidence indicates that LND cannot significantly improve the survival of patients with ICC. Because of the uncertain survival benefit, routine or preventive LND cannot be recommended at present. Further prospective randomized clinical trials remain necessary to address this issue.

## Author contributions

FL, YJ, and LJ contributed to conception and design of the study. FL organized the database. FL and XY performed the statistical analysis. FL wrote the first draft of the manuscript. QL, SH, JC, SY, YF and FL wrote sections of the manuscript. All authors contributed to manuscript revision, read, and approved the submitted version.

## Conflict of interest

The authors declare that the research was conducted in the absence of any commercial or financial relationships that could be construed as a potential conflict of interest.

## Publisher’s note

All claims expressed in this article are solely those of the authors and do not necessarily represent those of their affiliated organizations, or those of the publisher, the editors and the reviewers. Any product that may be evaluated in this article, or claim that may be made by its manufacturer, is not guaranteed or endorsed by the publisher.
